# The Importance of Nitric Oxide as the Molecular Basis of the Hydrogen Gas Fumigation-Induced Alleviation of Cd Stress on *Ganoderma lucidum*

**DOI:** 10.3390/jof8010010

**Published:** 2021-12-23

**Authors:** Dyaaaldin Abdalmegeed, Gan Zhao, Pengfei Cheng, Javaid A. Bhat, Wajid Ali Khattak, Mostafa G. Ali, Fawze Alnadari, Ilyas Ali, Qurban Ali, Sameh A. Korma, Yehia A.-G. Mahmoud, Manar K. Abd Elnabi, Weiti Cui, Wenbiao Shen

**Affiliations:** 1 Laboratory Center of Life Sciences, College of Life Sciences, Nanjing Agricultural University, Nanjing 210095, China; 2018116158@njau.edu.cn (D.A.); 2018216033@njau.edu.cn (G.Z.); 2020216037@njau.edu.cn (P.C.); wtcui@njau.edu.cn (W.C.); 2 Center of Hydrogen Science, Shanghai Jiao Tong University, Shanghai 200240, China; 3 Microbiology Section, Botany Department, Faculty of Science, Tanta University, Tanta 31527, Egypt; yehia.mahmoud@science.tanta.edu.eg (Y.A.-G.M.); manar_kamal@ymail.com (M.K.A.E.); 4 State Key Laboratory for Crop Genetics and Germplasm Enhancement, National Center for Soybean Improvement, Nanjing Agricultural University, Nanjing 210095, China; javid.akhter69@gmail.com; 5 Key Laboratory of Crop Physiology and Ecology, Ministry of Agriculture, Nanjing Agricultural University, Nanjing 210095, China; wajid_82@ymail.com; 6 Botany and Microbiology Department, Faculty of Science, Benha University, Benha 13511, Egypt; mustafa.abdelbary@fsc.bu.edu.eg; 7 Department of Food Science and Engineering, College of Food Science and Technology, Nanjing Agricultural University, Nanjing 210095, China; 2019208038@njau.edu.cn; 8 College of Animal Science and Technology, Nanjing Agricultural University, Nanjing 210095, China; 2017205030@njau.edu.cn; 9 Key Laboratory of Monitoring and Management of Crop Diseases and Pest Insects, Nanjing Agricultural University, Nanjing 210095, China; qurbanalirattar@webmail.hzau.edu.cn; 10 Department of Food Science, Faculty of Agriculture, Zagazig University, Zagazig 44511, Egypt; sameh.hosny@zu.edu.eg

**Keywords:** hydrogen fumigation, nitric oxide, hydrogen-rich water, stress alleviation, cysteine, proline, *Ganoderma lucidum*

## Abstract

Whether or not hydrogen gas (H_2_) can reduce cadmium (Cd) toxicity in *Ganoderma lucidum* has remained largely unknown. Here, we report that Cd-induced growth inhibition in *G. lucidum* was significantly alleviated by H_2_ fumigation or hydrogen-rich water (HRW), evaluated by lower oxidative damage and Cd accumulation. Moreover, the amelioration effects of H_2_ fumigation were better than of HRW in an optimum concentration of H_2_ under our experimental conditions. Further results showed that H_2_-alleviated growth inhibition in *G. lucidum* was accompanied by increased nitric oxide (NO) level and nitrate reductase (NR) activity under Cd stress. On the other hand, the mitigation effects were reversed after removing endogenous NO with its scavenger cPTIO or inhibiting H_2_-induced NR activity with sodium tungstate. The role of NO in H_2_-alleviated growth inhibition under Cd stress was proved to be achieved through a restoration of redox balance, an increase in cysteine and proline contents, and a reduction in Cd accumulation. In summary, these results clearly revealed that NR-dependent NO might be involved in the H_2_-alleviated Cd toxicity in *G. lucidum* through rebuilding redox homeostasis, increasing cysteine and proline levels, and reducing Cd accumulation. These findings may open a new window for H_2_ application in Cd-stressed economically important fungi.

## 1. Introduction

*Ganodermalucidum* is one of the large basidiomycetous fungi with a highly significant medicinal importance [1]. Nevertheless, the development of its commercial value is impeded by the limitations of its basic biological research. Cadmium (Cd) is one of the most harmful heavy metals due to its carcinogenic and neurotoxic effects on animals and humans [2]. Due to its high solubility in water, it can readily enter into different life forms, including microbes, plants, and animals [3]. Excess Cd accumulation inside living organisms can disrupt physiological processes such as protein metabolism, respiration, and nutrients uptake [4], as it induces the production of reactive oxygen species (ROS) [5,6]. ROS, in turn, oxidize vital biological molecules such as proteins, lipids, and nucleic acids, leading to membrane damage, inactivation of enzymes, and lipid peroxidation [5]. The mushrooms’ cultivation medium mainly consists of rice and wheat straw that might grow in soil contaminated with Cd [7]. So, *G. lucidum* accumulates Cd while consuming contaminated straw, leading to reduced mycelial growth and biomass. Furthermore, the contaminated mushrooms are finally eaten by humans, and their health is adversely affected.

Hydrogen gas (H_2_) application, commonly in the form of hydrogen-rich saline or hydrogen-rich water (HRW), has been utilized in medicine for the treatment of many diseases [8,9,10]. The first study was conducted on animals, wherein H_2_ efficacy was explained by its ability to reduce reactive oxygen species (ROS), mitigating the oxidative damage caused by ischemia/reperfusion injury in the rat brain [8]. Numerous studies followed this study in the medical field. Meanwhile, H_2_ was extensively applied in agriculture, mainly in the form of HRW, for enhancing plant tolerance to abiotic stresses such as drought [11] and heavy metals [12,13,14,15,16], as well as regulating plant growth and development [17,18,19]. In contrast to animals and plants, few studies concerning the effects of H_2_ on fungi were documented. The first study on fungi reported that HRW could ameliorate acetic acid stress by modulating the impact of ROS balance on morphology, growth, and secondary metabolism via enhancing glutathione peroxidase in *G. lucidum* [20]. Another study on the mushroom *Hypsizygus marmoreus* reported that, though strengthening the antioxidant system, HRW could alleviate the toxicities of NaCl, CdCl_2_, and H_2_O_2_ [21], improve the post-harvest quality, and reduce rot incidence [22].

The direct supply of H_2_ in its gaseous form, called fumigation, was rarely applied and was confined to H_2_-modified atmosphere studies, targeting fresh food storage [23,24,25,26] as well as soil treatment to enhance the plant growth via improving the rhizosphere micro-environment [27,28,29]. The concentration of H_2_ by direct gas supply is higher than when it is bubbled into water (HRW), and the hydrogen solubility and retention time in water are short, so HRW preparation technology needs improvements, particularly in agricultural applications [30]. Regarding mushrooms, there are additional problems concerning the HRW delivery method, such as the necessity for optimum pH and humidity levels, which are critical factors for fungal growth [31,32]. Therefore, HRW is an unsuitable form of H_2_ delivery for fungi, particularly for solid-state growth, because the water content in HRW would interfere with the humidity content and may alter the pH value in the culture environment. Moreover, the application of HRW on the solid-state culture is relatively inconvenient, unlike the H_2_ gas fumigation technique. Therefore, our research aims to overcome the possible HRW problems by directly utilizing H_2_ gas, which can spread rapidly and evenly in the cultivation area. Since H_2_ is neither flammable nor explosive at concentrations less than 4% [33], we examined different H_2_ gas fumigation concentrations within the safe range for its ability to alleviate the Cd-induced mycelial growth inhibition and regulate Cd accumulation in *G. lucidum* and compared its efficacy to the HRW’s.

Nitric oxide (NO) is another key signaling molecule that regulates several physiological and biochemical responses to abiotic stresses in animals, plants, and fungi. In fungi, it was reported that 6 h heat treatment raised the concentration of NO in *Trichoderma harzianum* mycelia [34]. Additionally, sodium nitroprusside (SNP), the NO-releasing compound, relieved the oxidative damage caused by heat stress in *Pleurotus eryngii var. tuoliensis* [35] and alleviated copper toxicity in *Saccharomyces cerevisiae* [36]. As a second messenger, NO was confirmed as a participant in many key signaling pathways in animals and plants. However, its role in microorganisms is still largely unknown [37].

The enhancement of the antioxidant system, and hence the regulation of redox homeostasis, is a well-known mechanism of the H_2_ alleviation effect under stresses in plants and fungi [30]. It was confirmed that the H_2_-induced NO acts upstream of the antioxidant system in higher plants under different stresses, such as drought [38] and Cd exposure [39], and participates in root organogenesis [18,40]; however, the possible link between H_2_ and NO in fungi is still unidentified. Thus, in the present study on the fungus *G. lucidum*, the early minute-scale time course of the endogenous H_2_ and NO signals was observed under Cd stress, and we found that H_2_ signal preceded NO signal. This gave us a clue that NO may be induced by H_2_ in fungi. So, exogenous H_2_ and/or NO were added under Cd stress to mimic the physiological effects of these molecules and examine their possible link through their effects on phenotype and physiology. Nitrate reductase (NR) is the well-known enzyme responsible for NO production in plants and fungi [41,42]. Additionally, it was reported that NO synthesis was regulated by NR during development in *Aspergillus nidulans* [43]. Therefore, the NO scavenger, cPTIO, and the NR inhibitor, sodium tungstate (Na_2_WO_4_), were administered after H_2_ in our study to investigate the NO’s involvement in the H_2_-induced responses.

Cysteine (Cys) was demonstrated to effectively reduce Cd toxicity in Arabidopsis [44]. Previous studies suggested the role of Cys in the alleviation of stresses via the enhancement of the glutathione (GSH) system and the stimulation of cell proliferation in humans [45,46]. The availability of Cys is a limiting factor for glutathione biosynthesis [47,48]. GSH is a major non-enzymatic antioxidant and precursor of phytochelatins, which have metal binding affinity [49]. Cys could enhance chromium tolerance by enhancing GSH generation and heavy metal chelation in *Arabidopsis* [50]. Recently, Cys has been reported to act downstream of NO, mitigating chromium toxicity in tomato seedlings [51]. The report on fungi demonstrated that exogenous Cys treatment participated in the regulation of morphogenesis and the mitochondrial activity of *Histoplasma capsulatum* [52]. Similarly, proline is another significant amino acid that was revealed to participate in the stress response of different organisms [53]. It was reported that proline accumulation was enhanced by adding exogenous NO under osmotic stress in wheat seedlings [54]. However, the corresponding role of cysteine and proline in fungi is still unclear.

Previous research suggested a link among H_2_, NO, some amino acids such as cysteine and proline, as well as their effects on stress alleviation. Thus, in this study, we examined the role of NO in H_2_-alleviated Cd stress in *G. lucidum* and further explored the mediation role of cysteine and proline. More importantly, it provides new ideas for improving the production of economically important fungi.

## 2. Materials and Methods

### 2.1. Chemicals

Unless stated otherwise, all the chemicals used in this study were purchased from Sigma-Aldrich (St Louis, MO, USA). Cadmium chloride (CdCl_2_, CAS 10108-64-2) was purchased from Shanghai Aladdin Biochemical Technology Co., Ltd. The concentrations of the following chemicals were determined in pilot experiments. Hydrogen gas (purity ≥ 99.99%) was prepared by a generator (SHC-300, Saikesaisi Hydrogen Energy Co., Ltd., Jinan, Shandong, China). The chemicals utilized in our treatments were: sodium tungstate (Na_2_WO_4_; 500 μM, CAS 10213-10-2, purity 99%), an inhibitor of nitrate reductase, the enzyme responsible for NO formation [55]; sodium nitroprusside (SNP; 500 μM, CAS 13755-38-9, purity 98%), a well-known NO-releasing compound [56,57]; 2-(4-carboxyphenyl)-4,4,5,5-tetramethylimidazoline-1-oxyl-3-oxide potassium salt (cPTIO; 500 μM, CAS 148819-94-7; purity 97%), a well-known NO scavenger [41,58,59]; and proline (10 μM, CAS 147-85-3, purity 99%), an antioxidant. Moreover, cysteine was used at 300 µM and GSH at 325 µM. The GSH synthesis inhibitor _DL_-buthionine-[*S*,*R*]-sulfoximine (BSO) was used at 2.5 mM. To ensure NO’s role, old SNP was used as a negative control and was prepared by keeping 500 μM SNP solution in an open tube in the light for 10 d to eliminate the entire NO [60].

### 2.2. Preparation of Hydrogen-Rich Water (HRW)

The procedures were carried out according to the previously mentioned method [60], with slight modifications. The H_2_ gas generator (SHC-300, Saikesaisi Hydrogen Energy Co., Ltd., Shandong, China) was utilized to produce hydrogen gas (99.99%, *v*/*v*). To prepare saturated HRW, the H_2_ gas generated was bubbled into 1000 mL of sterile water at a rate of 150 mL per minute for 30 min. The concentration of H_2_ at saturation point was 0.8 mM. Subsequently, 10, 25, 50, 75, or 100 mL of the saturated HRW was immediately mixed with 90, 75, 50, 25, or 0 mL ddH_2_O, respectively, to prepare 10%, 25%, 50%, 75%, or 100% HRW concentrations (*v*/*v*). Freshly prepared HRW was pre-sterilized through a 0.22 µM membrane before application to mycelia. The mycelia plates were treated with 15 mL for each treatment, and 15 mL pre-sterilized ddH_2_O was added to the control plates.

### 2.3. Fungal Materials, Growth Condition, and Screening of Cd Inhibitory Concentrations

The wild-type strain (*Ganoderma lucidum* HG) was obtained from the Edible Fungi Institute, Shanghai Academy of Agricultural Science, China. Potato dextrose agar (PDA) was used as a culture medium for the strains of *G. lucidum* incubated at 28 °C in the dark for 7 d (solid seed). From the solid seed, 6 mm-diameter mycelial discs were inoculated into solid complete yeast medium (CYM) plates (2% glucose, 1% maltose, 0.05% MgSO_4_·7H_2_O, 0.2% yeast extract, 0.2% tryptone, 0.46% KH_2_PO_4_, 1% agar, initial pH = 5.5), supplied with CdCl_2_ at different concentrations, 0, 1, 1.5, 1.75, and 2 mM. The cultures were incubated in the dark at 28 °C for 8 d; then, the mycelial growth diameters were immediately measured. Meanwhile, eight mycelial discs, each of 6 mm diameter, were cut from the same growth diameter at the edge of the solid seed colony. Each set of 8 mycelial discs was grown on 100 mL liquid CYM media containing different concentrations of CdCl_2_ 0, 50, 100, 200, 300, and 400 μM. The cultures were incubated in shaking incubator in the dark at 28 °C for 8 d. Afterward, the mycelia were harvested and washed with distilled water and then oven-dried at 60 °C to a constant biomass, and the mycelial biomass dry weight values were instantly determined.

### 2.4. Experimental Design: Response of G. lucidum to Different Concentrations of H_2_ Fumigation

*G. lucidum* mycelia were grown on control and stressed CYM Petri-plates, which were let open in pre-sterilized plastic containers (10.5 L, Lock & Lock, Suzhou, China), sealed with vaseline. Fumigation of *G. lucidum* with H_2_ was carried out in the plastic containers according to the previous method [23], with slight modifications. An airbag with a sampling plug was filled with H_2_ from the H_2_ gas generator. Gas syringes (Hamilton Co., Reno, NV, USA) were used for H_2_ gas sampling from the airbag and then were used to inject H_2_ through 0.22 µM sterilization filter that ends with a sterilized needle into the fumigation containers. Mycelia were incubated in a hydrogen-containing atmosphere at 28 ± 1 °C for 24 h. Another set of non-fumigated mycelia was incubated under the same conditions as control.

Then, 6 mm-diameter mycelial discs were cut from a seven-day-old *G. lucidum* (solid seed), and each disc was inoculated into one of the solid CYM plates. Then, the culture plates were aseptically transferred into the fumigation containers and grown in the dark for 2 days at 28 °C. On the 3rd day, mycelia were either pre-treated with different H_2_ fumigation concentrations, 1%, 2%, 3%, 5%, or 10%, or with 50% HRW, each for 24 h, twice, once every 12 h, or not treated with either hydrogen forms and used as control (Con). On the 4th day, 6 mm-diameter mycelial discs from H_2_- or HRW-treated or non-treated mycelia were cut and grown on plain solid CYM (−Cd) or 1.75 mM CdCl_2_-containing solid CYM (+Cd) for 8 d at 28 °C in the dark; then, the diameters of fungal growth were measured. Simultaneously, the effect of H_2_ on the mycelial biomass dry weight was determined by cutting 6 mm-diameter mycelial discs on the 4th day from H_2_- or HRW-treated or non-treated mycelia from the same growth diameter. Then, each set of 8 mycelial discs was inoculated into 100 mL plain liquid CYM (−Cd) or 200 μM CdCl_2_-containing liquid CYM (+Cd) and grown for 8 d in shaking incubator at 28 °C in the dark. Afterward, the mycelia were harvested and washed with distilled water and then oven-dried at 60 °C to a constant biomass. The biomass dry weight values were immediately measured. Additionally, mycelial samples were collected and immediately used or stored at −80 °C for further analysis.

To address the possible oxygen deficiency that might occur in the fumigation containers, in our preliminary trials, the mycelial growth in the plates inside the containers was compared to that in parafilm-sealed plates that were not enclosed inside containers, under the same incubation conditions, and we found no phenotypic difference between them. Additionally, the containers were recurrently opened under sterilized conditions for 15 min across a 12 h interval. Moreover, 10 g calcium hydroxide Ca(OH)_2_, the CO_2_ absorbent, was placed in the containers to absorb the excess CO_2._

### 2.5. Determination of Intracellular Cadmium Content

Cd content in the fungal mycelia was assessed according to the previous method [61], with a slight modification. Briefly, mycelia were washed with distilled water to remove the non-specific bound Cd. Then, mycelia were dried in the oven at 60 °C till constant weight. Afterward, 0.2 g dry samples were ground to a fine powder and then mixed with 6 mL of HNO_3_ and 2 mL of H_2_O_2_. The mixture was digested via microwave digestion (Milestone Ethos T). The digested sample was transferred into a quartz crucible at 60 °C for 45 min to evaporate excess reagents. ddH_2_O was used to complete the sample volume to 10 mL, and inductively coupled plasma optical emission spectroscopy (ICP-OES; PerkinElmer Optima 2100DV) was utilized to determine the Cd content in the samples. Three replicas for each sample were prepared. The parameters were set according to the previous method [61]. A range of Cd concentrations was measured to generate a standard curve for Cd.

### 2.6. Effect of H_2_ Fumigation on Reactive Oxygen Species (ROS) and TBARS Contents

The detection of ROS fluorescence intensity was carried out according to the previous method [62]. The pre-sterilized glass coverslips that had been inserted in the solid CYM media and had mycelial growth on their surface were picked and stained with 2, 7 dichlorodihydrofluorescein diacetate (DCHF-DA; 10 mM phosphate-buffered saline (PBS) (pH 7.5)) for 20 min in the dark. Then, the hyphae were gently washed with 10 mM PBS (pH 7.5) to remove the excess stain. Confocal laser microscope (TCS SP2; Leica) at 488 nm emission wavelength was utilized to visualize the ROS fluorescence produced by an argon laser and a 525 to 530 nm filter. The fluorescence intensity was analyzed by ZEN lite (Zeiss software).

TBARS content, the indicator of lipid peroxidation, was measured using the previously described method [63], with a slight modification. A small amount of liquid nitrogen was added to 0.1 g of mycelia before grinding it. After that, 2 mL of 5% TCA (CAS 76-03-9; purity 99%) was added and then centrifuged at 10,000× *g* at 4 °C for 10 min. Then, 2 mL of the supernatant was collected and added to 2 mL 0.67% 2-thiobarbituric acid (TBA; CAS 504-17-6; purity 98%). The mixture was inserted into a boiling water bath and exposed to heat for 15 min. Afterward, the sample was centrifuged at 10,000× *g* for 10 min at 4 °C. The supernatant was collected, and its level of absorbance was quantified at 532 nm. The results obtained were corrected for non-specific absorption by deducting the reading of absorbance at 600 nm. The levels of TBARS were estimated using the extinction coefficient of 155 mM^−1^ cm^−1^.

### 2.7. Measurement of the Antioxidant Enzyme Activities

The following procedures were followed to determine the activities of different enzymes. Mycelial samples were frozen and ground in liquid N_2_. Briefly, 0.3 g of frozen mycelia were minced into a uniform consistency using liquid N_2_ and 5 mL of potassium phosphate-buffered solution (50 mM, pH 7.0). Then, the homogenate was centrifuged at 15,000× *g* for 20 min at 4 °C, and the resulting supernatant was instantly utilized for the following enzyme assays. Protein contents were measured according to Bradford method using bovine serum albumin (BSA) as a standard [64].

Catalase (CAT) activity was evaluated by determining the decomposition rate of hydrogen peroxide (H_2_O_2_) at 240 nm according to the previous procedures [65]. The assay of SOD activity was carried out according to the previous method [66] by tracking the inhibition of the photochemical reduction in nitro blue tetrazolium (NBT). One unit of SOD activity was represented as the amount of enzyme consumed to produce 50% of NBT reduction inhibition, observed at 560 nm. Glutathione Peroxidase Cellular Activity Assay Kit (Beyotime Institute of Biotechnology) was used to measure glutathione peroxidase (GPX) activity. During the assay, GPX in the supernatant oxidizes GSH to GSSG, and this reaction is coupled to the recycling of GSSG back to GSH via glutathione reductase (GR), consuming NADPH. The decrease in the absorbance of NADPH at 340 nm is the indicator of GPX activity, since the GPX is the factor limiting the rate of the coupled reactions. GR activity was detected using an assay kit (Beyotime Institute of Biotechnology) by monitoring the decrease in absorbance at 340 nm.

### 2.8. Detection of Endogenous H_2_

Endogenous H_2_ content in *G. lucidum* mycelia was measured according to the previous method [67], with some modifications. Headspace sampling of H_2_ was followed by gas chromatography (GC). Mycelia (0.3 g) were homogenized for 1 min, placed in a vial, and then 7 mL ddH_2_O, 5 mL octanol (purity 99%), and 0.5 mL sulfuric acid (5 M, purity 99.9%) were added. Afterward, nitrogen gas was added to the vial to replace the air. Afterward, the vial was instantly capped and shaken strongly by hand for 1 min. Finally, the vials were heated for 1 h at 70 °C to liberate H_2_ gas from the fungal mycelia and then left to cool at room temperature before GC analysis. The chromatographic system and its parameters were according to the previous method [67].

### 2.9. Detection of Nitrate Reductase (NR) Activity

Nitrate reductase assay was carried out according to the previous method [68]. The nitrite produced was determined by spectrophotometry at 540 nm. The results were represented as the formation of nmol of nitrite/min/mg protein.

### 2.10. Detection of Nitric Oxide by Confocal Laser Microscopy and Spectrophotometry

Nitric oxide content was determined by the fluorescent probe diaminofluorescein-FM diacetate (DAF-FM DA; CAS number 254109-22-3, purity ≥ 98%) according to the previous methods [43]. Staining of mycelia was carried out for 20 min with DAF-FM DA to visualize nitric oxide. The fluorescence was detected by a confocal laser microscope with excitation at 488 nm, and ZEN lite (Zeiss software) was used to analyze the fluorescence intensities. NO contents were also measured by spectrophotometric Griess reagent method, with slight modification according to [59].

### 2.11. Measurement of Proline, Cysteine, and Glutathione Contents

Proline contents were analyzed according to the previous procedures [69] with minor modifications. Briefly, the quantification of proline was carried out by its derivatization with phenylisothiocyanate, and a reversed-phase HPLC (HITACHID-2000, Tokyo, Japan) was used to separate the resulting derivatives. Cysteine and glutathione levels were detected according to the method previously described [70].

### 2.12. Determination of Gene Expression in G. lucidum

The expression levels of the indicated genes in *G. lucidum* were determined by RT-qPCR according to the previous procedures [71]. The sequences of the primers used in the gene analysis and the reference gene are listed in Supplementary Table S1.

### 2.13. Statistical Analysis

Data are the means ± SD of three independent experiments with three replicates at least for each. For data analysis, a *t*-test (*p* < 0.05) or multiple-comparison one-way analysis of variance (ANOVA) followed by Tukey’s test (*p* < 0.05) was chosen depending on the experiment type.

## 3. Results

### 3.1. H_2_ Fumigation Alleviated Cd-Induced Inhibition of Mycelial Growth Diameter and Biomass Dry Weight in Ganoderma lucidum Mycelia

In our experiments, the mycelia of *G. lucidum* grown on solid CYM media were treated with different concentrations of CdCl_2_: 0, 1, 1.5, 1.75, and 2 mM (Supplementary Figure S1A). Among them, 1.75 mM CdCl_2_ resulted in about 50% inhibition in the mycelial growth diameter in the solid culture. However, in the liquid culture, 200 µM CdCl_2_ caused about 50% inhibition of the biomass dry weight (Supplementary Figure S1B). Therefore, 1.75 mM or 200 μM CdCl_2_ in solid or liquid cultures, respectively, were selected for all subsequent stress treatments. Additionally, our results showed that 50% HRW pre-treatment elicited the highest alleviation in diameter and dry weight of the stressed mycelia (Supplementary Figure S2). So, 50% HRW was used as a positive control in the subsequent H_2_ fumigation experiments.

**Figure 1:**
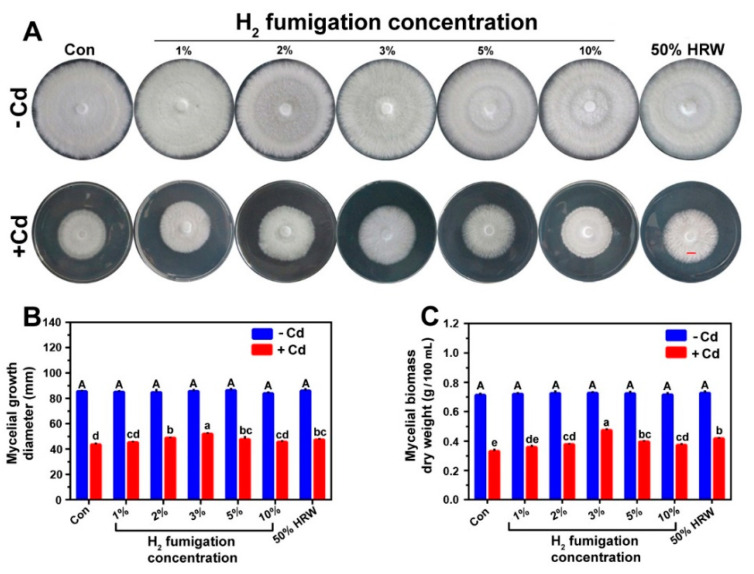
H_2_ fumigation increased the mycelial growth diameter and the biomass dry weight of *G. lucidum*. Mycelial inocula were grown on solid CYM media for 2 days; then, on the 3rd day, were either pre-treated with 1%, 2%, 3%, 5%, or 10% H_2_ fumigation or 50% HRW for 24 h or not treated with either hydrogen forms (Con). On the 4th day, 6 mm-diameter mycelial discs from different treatments were cut and grown on solid CYM with or without CdCl_2_ for 8 d; then, photographs of the plates were taken (**A**). Scale bar = 1 cm. Diameters of mycelial growth were detected (**B**). Simultaneously, on the 4th day, mycelial discs of 6 mm-diameter were cut from H_2_- or HRW-treated or non-treated mycelia. Each set of 8 mycelial discs was inoculated into liquid CYM media with or without CdCl_2_ then grown in shaking incubator for 8 days. Then, the biomass dry weight values were immediately determined (**C**). Bars with different letters are significantly different at *p* < 0.05 according to one-way ANOVA with multiple comparison using Tukey’s test.

To determine the most effective H_2_ fumigation concentration that significantly alleviated the Cd-caused growth inhibition, another experiment was performed in which the mycelia were grown in CYM plates and were pre-treated with the H_2_ fumigation concentrations 1%, 2%, 3%, 5%, or 10% or with 50% HRW (as a positive control) before exposure to Cd stress. As shown in Figure 1A–C, among the H_2_ concentrations, 2%, 3% (in particular), and 5% showed significant alleviation of the inhibition caused by Cd stress, whereas lower and higher concentrations brought about weaker responses. The maximum mitigation effect on mycelial growth diameter and biomass was observed at 3% H_2_ concentration, with a higher alleviation effect than the 50% HRW pre-treatment.

### 3.2. H_2_ Fumigation Reduced Cd Accumulation, Decreased the Production of ROS and Alleviated Lipid Peroxidation in G. lucidum under Cd Stress

**Figure 2:**
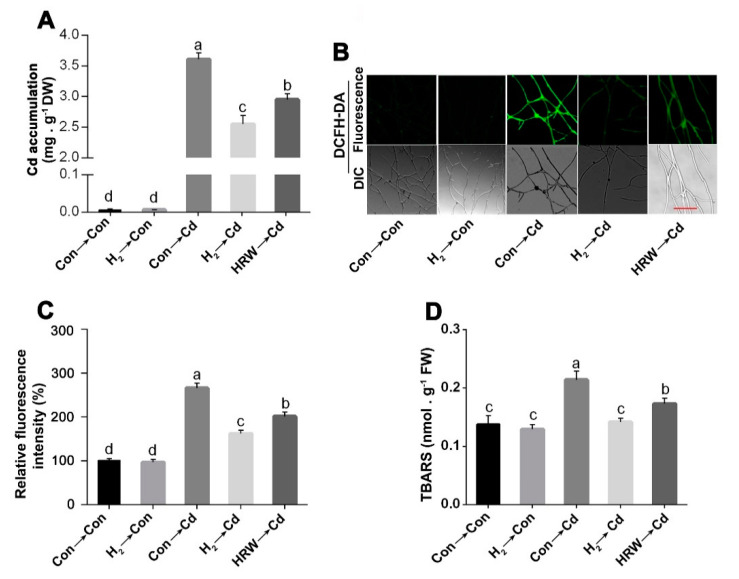
H_2_-fumigation pre-treatment reduced Cd accumulation and decreased ROS and TBARS contents in *G. lucidum*. Mycelia were cultured on solid CYM media for 2 days. Then, on the 3rd day, mycelia were pre-treated with 3% H_2_ fumigation, 50% HRW for 24 h, or not treated with either hydrogen form (control). On the 4th day, 6-mm-diameter mycelial discs from these treatments were transferred to solid CYM or 1.75 mM CdCl_2_-containing solid CYM, each with or without inserted glass coverslips then grown for 5 days. Afterward, samples from the plates without coverslips were immediately used for Cd content analysis (**A**). Coverslips with mycelia on their surface were loaded with DCFH−DA, and then the ROS fluorescence intensities were analyzed via confocal laser microscopy (**B**). Scale bar = 100 µm. DIC, differential interference contrast. Respective fluorescence intensities were measured (**C**). Mycelia were also sampled for TBARS analysis (**D**). Mycelia without chemicals were used as a control for each analysis (Con → Con). Bars with different letters are significantly different at *p* < 0.05 according to one-way ANOVA with multiple comparison using Tukey’s test.

As shown in Figure 2A, the mycelia accumulated Cd when treated with 1.75 mM CdCl_2_ for 24 h. Cd accumulation in the mycelia pre-treated with 3% H_2_ fumigation showed a significant decline by 29.13% compared to Cd stress alone mycelia. The reduction in Cd accumulation by H_2_ gas fumigation was more pronounced than that achieved by 50% HRW, suggesting the higher efficacy of H_2_ fumigation relative to HRW. This is in agreement with a previous study that showed that HRW significantly alleviated mycelial growth inhibition under acetic acid stress in *G. lucidum* but did not affect it under normal conditions [20].

The effect of H_2_ fumigation on the ROS production caused by Cd stress was detected by loading the mycelia with 2′, 7′ dichlorofluorescein diacetate (DCFH-DA) probe. A sharp decline was detected in the DCFH-DA fluorescence intensity in the H_2_-fumigated mycelia by 39.63% compared to Cd alone sample (Figure 2B,C). Moreover, the redox alleviation in the H_2_-fumigated mycelia was more evident than HRW-treated mycelia, which exhibited a decrease in DCFH-DA fluorescence intensity by only 24.15%. These findings suggested the advantages of H_2_ fumigation over HRW in maintaining redox homeostasis under Cd stress. We also noticed that H_2_ fumigation did not significantly alter the ROS accumulation under normal conditions.

The intracellular TBARS contents were also measured to detect the impact of Cd and H_2_ on lipid peroxidation. As shown in Figure 2D, despite no significant difference in the TBARS content between the H_2-_treated and non-treated mycelia under normal conditions, there was a marked decline in TBARS contents in the H_2_- and Cd-treated mycelia than those samples with only Cd stress, reaching a similar level of unstressed control.

### 3.3. H_2_ Fumigation Enhanced the Antioxidant Enzymes’ Activities and Their Corresponding Transcripts under Oxidative Stress Caused by Cd

To understand the mechanism by which H_2_ achieved redox homeostasis, we detected the activities of the antioxidant enzymes: catalase (CAT), superoxide dismutase (SOD), glutathione reductase (GR), and glutathione peroxidase (GPX). Our results showed that, under Cd stress, the activities of CAT, GR, and GPX were decreased by 55.40%, 46.43%, and 43.67%, respectively; however, SOD activity was increased by 24.31%. Interestingly, in the H_2_-pretreated Cd-stressed mycelia, all these antioxidant enzyme activities were significantly increased relative to the Cd stress alone of mycelia. Moreover, the transcript levels of the related genes were further quantified by RT-PCR. Gene expression levels of *cat1/2*, *sod1/4*, *gr*, and *gpx* showed similar tendencies to the corresponding antioxidant enzyme activities (Supplementary Figure S3).

### 3.4. Endogenous NO Was Involved in the H_2_-Induced Cd Stress Tolerance

**Figure 3:**
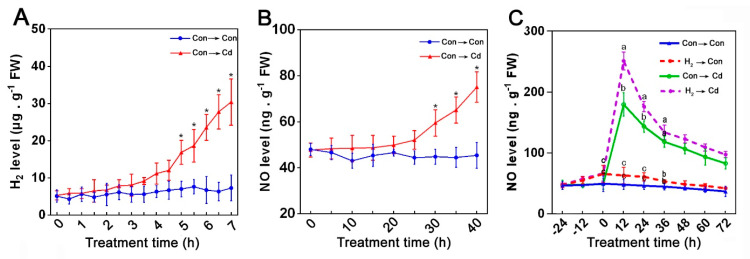
Effects of Cd and H_2_ fumigation treatments on the endogenous H_2_ and NO levels in mycelia. Early signals of H_2_ and NO were observed in the mycelia grown on solid CYM with or without 1.75 mM Cd for the indicated time points (**A**,**B**). Additionally, a four-day time course of endogenous NO level was tracked in the mycelia (**C**). On the 3rd day, two-day-old mycelia were either pre-treated or not with 3% H_2_ for 24 h and then were transferred into control or stressed CYM media and grown for 72 h. H_2_ and NO levels were measured by GC and Griess reagent assay, respectively. Asterisks * indicate the significant difference at every time point relative to Con → Con according to *t*-test at *p* < 0.05 (**A**,**B**), and letters indicate significant difference at every time point relative to Con → Con according to one-way ANOVA with multiple comparison using Tukey’s test at *p* < 0.05 (**C**).

To know whether or not NO proceeded as a downstream signal of H_2_ in the Cd tolerance in *G. lucidum*, the early signals of H_2_ and NO were observed. The endogenous H_2_ content increased steadily in the Cd-treated mycelia with the time extension, and it showed a marked difference between control and Cd stress only after 5 min (Figure 3A). However, the NO content in the Con- and Cd-treated mycelia had a slight difference during the first 20 min and then increased significantly in the Cd-stressed mycelia after 30 min (Figure 3B), suggesting a possible inherent role of the endogenous H_2_ in enhancing NO production in *G. lucidum* under Cd stress. Thus, we investigated the effect of H_2_ fumigation on the endogenous NO content under stress and control conditions across a 4-day time course (Figure 3C), and we found that, in comparison to the control samples, the mycelia treated with Cd alone exhibited a rapid burst of NO release at 12 h and then declined until 72 h. Moreover, the Cd-induced NO production in the mycelia was markedly enhanced by H_2_ fumigation relative to the Cd alone treated samples. Moreover, H_2_-fumigation for 24 h also led to a slight NO accumulation in the control samples. These results gave us a clue about NO’s importance in the H_2_-mediated alleviation of Cd stress in *G. lucidum*.

### 3.5. H_2_-Induced Alleviation of the Cd-Caused Mycelial Growth Inhibition and Excess Cd Accumulation Was Reversed by the NO Scavenger, cPTIO

**Figure 4:**
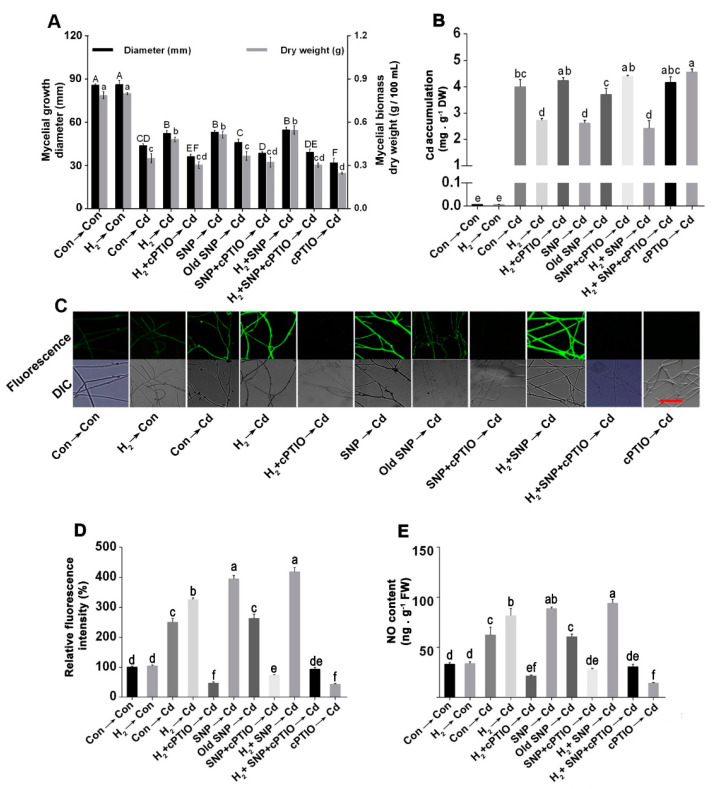
H_2_-enhanced NO production was susceptible to the NO scavenger cPTIO. Two-day-old mycelia were either pre-treated or not with 3% H_2_ fumigation for 24 h. In all tests of this experiment, stressed mycelia were either treated or not with 500 µM SNP, 500 µM old SNP, or 500 µM cPTIO alone or in the indicated combinations for 30 min after H_2_ pre-treatment. On the 4th day, 6-mm-diameter mycelial discs from the different treatments were cut and grown for 8 d on solid CYM with or without 1.75 mM CdCl_2_ for growth diameter observation (**A**; left) or liquid CYM with or without 200 µM CdCl_2_ for biomass dry weight detection (**A**; right). Simultaneously, on the 4th day, mycelial discs from the different treatments were grown for 12 h or 5 days on stressed solid CYM. Five-day-stressed or non-stressed mycelia were sampled for Cd accumulation measurement (**B**). Twelve-hour-stressed or non-stressed mycelia were utilized for NO analysis via confocal laser microscopy (**C**). Scale bar = 100 μm. The respective fluorescence intensities were analyzed (**D**). NO contents were also measured by Griess reagent assay (**E**). Mycelia without chemicals were used as the control for each analysis (Con → Con), while the H_2_ alone treated mycelia were represented as H_2_ → Con. Bars with different letters are significantly different at *p* < 0.05 according to one-way ANOVA with multiple comparison using Tukey’s test.

To further test the possibility of NO involvement in the H_2_-elicited response under Cd stress, the NO scavenger cPTIO was used to eliminate the endogenous NO. Interestingly, the H_2_-induced responses were severely diminished by cPTIO, indicating a potential participation of NO in the H_2_-induced amelioration of the Cd-elicited growth inhibition. On the contrary, by adding the NO-releasing compound SNP, Cd-induced inhibition of mycelial growth diameter (Figure 4A; left) and biomass dry weight (Figure 4A; right) were ameliorated, while old SNP, the negative control of SNP, failed to induce such responses. These results demonstrated the signifi-cance of NO in the fungus tolerance against Cd stress. Moreover, further mitigation was observed when exogenous NO was administered after H_2_, suggesting a possible link between the two signaling molecules in the stress tolerance.

For further examination of the NO’s role in Cd stress alleviation, Cd accumulation was also analyzed (Figure 4B). A remarkable reduction in Cd accumulation was detected in the H_2_ and/or NO treated mycelia, and the highest alleviation was achieved under H_2_ and NO successive treatments. However, reversion of these responses occurred after combining treatment with cPTIO. The treatment with cPTIO alone before Cd exposure caused considerably higher Cd accumulation relative to that in the Cd alone treatment. Moreover, the administration of old SNP exhibited no significant response. These results corroborated the contribution of NO in the H_2_-induced reduction in Cd accumulation.

Furthermore, NO’s participation was confirmed by quantifying its contents in the mycelia by confocal laser microscopy (Figure 4C), and the respective fluorescence intensities were analyzed (Figure 4D). Relative to control mycelia, Cd-stressed mycelia exhibited higher NO contents. However, mycelia showed markedly higher NO contents when pre-treated with H_2_ and/or SNP under Cd stress. Furthermore, the H_2_ and/or NO-triggered response in the stressed mycelia was prevented by cPTIO. When old SNP was added before Cd stress, it did not alter NO level, while cPTIO pre-treatment caused a significant depletion in the NO level. Analogous results in NO content were observed using spectrophotometric Griess reagent analysis (Figure 4E).

### 3.6. The H_2_-Induced Cd Tolerance Was Linked to the NR-Dependent NO Formation

**Figure 5:**
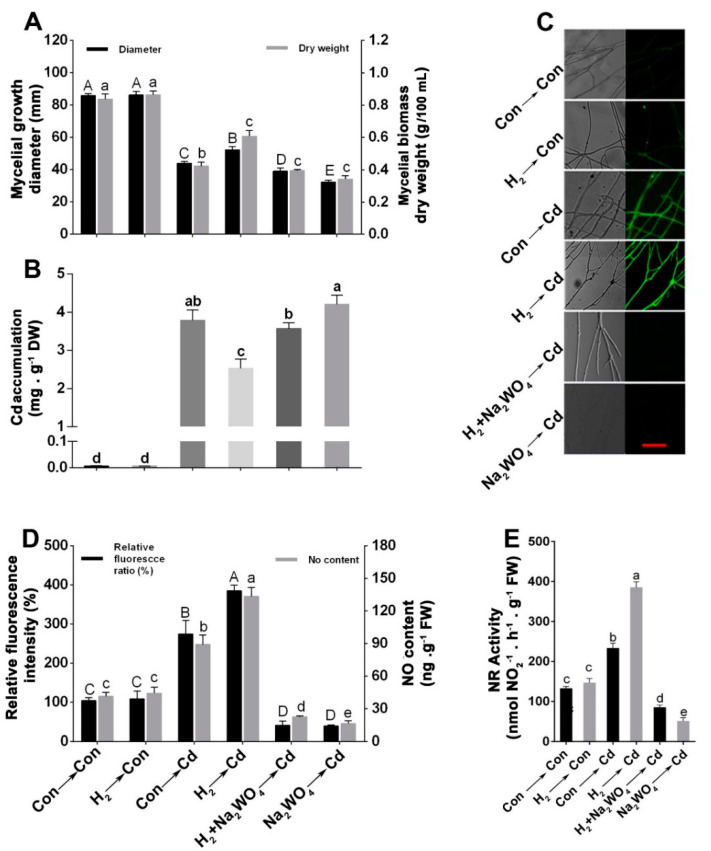
H_2_-induced NO production was sensitive to the NR inhibitor, Na_2_WO_4_. Mycelia were cultured on solid CYM media for 2 days. Then, on the 3rd day, they were either pre-treated or not with 3% H_2_ fumigation for 24 h. In all tests of this experiment, stressed mycelia were either treated or not with 500 μM Na_2_WO_4_ for 30 min after H_2_ pre-treatment. On the 4th day, 6-mm-diameter mycelial discs were cut from H_2_ treated or non-treated mycelia and grown for 8 d on solid CYM with or without 1.75 mM CdCl_2_ for growth diameter observation (**A**; left) or liquid CYM with or without 200 µM CdCl_2_ for biomass dry weight detection (**A**; right). Simultaneously, on the 4th day, mycelial discs from H_2_ treated or non-treated mycelia were grown for 12 h or 5 days on solid CYM with or without 1.75 mM CdCl_2_. Five-day-stressed or non-stressed mycelia were sampled for Cd accumulation measurement (**B**). Twelve-hour-stressed or non-stressed mycelia were used for NO analysis via confocal laser microscopy (**C**). Scale bar = 100 μm. The respective fluorescence intensities were measured (**D**; left). Contents of NO were measured by Griess reagent assay as well (**D**; right). Twelve-hour-stressed samples were also sampled for NR activity detection (**E**). Mycelia without chemicals were used as a control for each analysis (Con → Con), while H_2_ alone treated mycelia were represented as H_2_ → Con. Bars with different letters are significantly different at *p* < 0.05 according to one-way ANOVA with multiple comparison using Tukey’s test.

The source of NO in *G. lucidum* was further investigated by determining the impact of sodium tungstate (Na_2_WO_4_), an inhibitor of NR. A significant reduction in the mycelial growth diameter (Figure 5A; left) and the biomass dry weight (Figure 5A; right) was observed when Na_2_WO_4_ was applied. Interestingly, Na_2_WO_4_ addition markedly blocked the alleviation role of H_2_ in the Cd-elicited growth inhibition. Similarly, the H_2_-induced Cd accumulation decrease was reversed by Na_2_WO_4_ (Figure 5B). Na_2_WO_4_ pre-treatment alone showed higher Cd accumulation in the Cd-stressed mycelia.

The images of confocal laser microscopy exhibited that Cd- and/or H_2_-induced NO levels were suppressed by Na_2_WO_4_ (Figure 5C), which verified the NR source of NO under Cd and/or H_2_ treatments. Furthermore, results from corresponding fluorescence intensities (Figure 5D, left) and spectrophotometric analysis of NO contents (Figure 5D, right) suggested the potent inhibition of endogenous NO generation by Na_2_WO_4_ in mycelia. Furthermore, NR activity was determined (Figure 5E), and the results were consistent with NO contents. Therefore, these results demonstrate that the magnification of NO content by H_2_ and Cd was NR-dependent, and this further suggested that H_2_-triggered amelioration of Cd stress is possibly mediated by NR-related NO production. To minimize artefacts, mycelia were treated with H_2_, HRW, SNP, NO’s scavenger, cPTIO or NO’s inhibitor, and Na_2_WO_4_ alone or in the indicated combinations to investigate whether or not these chemicals have some effect that interferes with the response elicited by Cd stress in the combined treatment of Cd with these chemicals. This experiment showed no significant effect of either of these treatments on the mycelial growth or NO content under normal unstressed conditions (Supplementary Figure S7), consistent with our previous experiments that demonstrated no significant effect of the upstream signal (H_2_) under normal conditions.

### 3.7. Cysteine Was Involved in the H_2_-Induced, NO-Mediated Cd Tolerance

**Figure 6:**
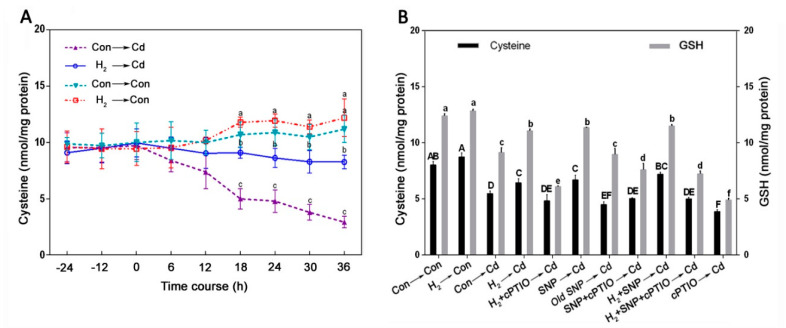
Time course of cysteine (Cys) content under H_2_ and Cd treatments and its sensitivity to NO scavenging. Two-day-old mycelia were either pre-treated or not with 3% H_2_ for 24 h and then were stressed or not with Cd for 36 h, and the Cys content was quantified at the indicated time points (**A**). Additionally, as shown in (**B**), two-day-old mycelia were either pre-treated or not with H_2_ for 24 h and then were stressed or not with Cd for 18 h. Stressed mycelia were either pre-treated or not with 500 μM SNP, 500 μM old SNP, or 500 μM cPTIO alone or in the indicated combinations for 30 min after H_2_ treatment. Afterward, Cys (**B**; left) and GSH (**B**; right) contents were measured. Different letters indicate significant differences between treatments at the indicated time points in A or after 18 h in B according to one-way ANOVA with multiple comparison using Tukey’s test at *p* < 0.05.

To address the underlying mechanism, time-dependent cysteine (Cys) accumulation was analyzed under H_2_ and Cd treatments. We noticed that, under Cd alone treatment, Cys levels were swiftly reduced through 36 h of Cd stress, while H_2_ pretreatment rescued the Cd-elicited drop in Cys content, especially after 18 h of Cd stress (Figure 6A). The role of Cys as a downstream component of NO signal in the alleviation of plant stresses was documented [51,72]. Thus, we further examined the possible involvement of NO in the H_2_-induced enhancement of Cys under Cd stress. Interestingly, pre-treatment with H_2_ and/or SNP resulted in the recovery of the Cd-elicited Cys decline. However, these responses were prevented by the treatment with cPTIO plus H_2_. We also noticed that old SNP did not ameliorate Cys level, and cPTIO pre-treatment caused further depletion of Cys (Figure 6B; left). Interestingly, similar trends were observed in the GSH levels under the same treatments (Figure 6B; right).

The effect of exogenous Cys on mycelial growth under Cd stress was also investigated, and the exogenous Cys-elicited response was compared to that of exogenous GSH. Exogenous Cys mitigated the inhibition of mycelial growth caused by Cd. However, this alleviation effect was significantly reduced in the samples pre-treated with BSO. Moreover, the treatment with BSO alone resulted in further depletion in the mycelial growth (Supplementary Figure S4A). Meanwhile, the evaluation of GSH levels was also performed. GSH levels were decreased under Cd stress and could be alleviated by exogenous Cys application (Supplementary Figure S4B). In addition, Cd accumulation was also measured under exogenous Cys treatment, and we observed that exogenous Cys markedly alleviated Cd accumulation in *G. lucidum*. However, the effects of exogenous Cys were weakened in the mycelia pre-treated with BSO (Supplementary Figure S4B).

### 3.8. H_2_-Enhanced Proline Content Was Susceptible to cPTIO

**Figure 7:**
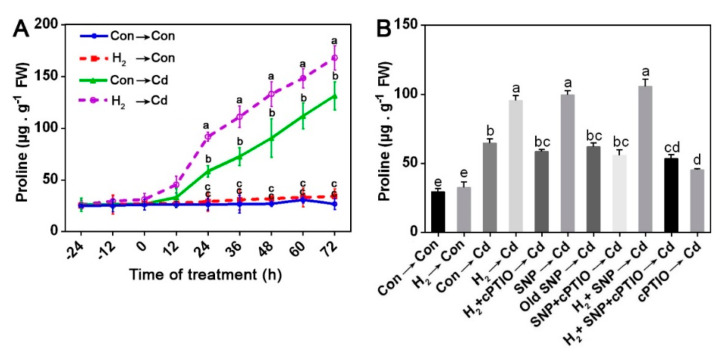
Time course of proline content under H_2_ treatment and its sensitivity to NO scavenging. Two-day-old mycelia were either pre-treated or not with 3% H_2_ for 24 h and then were stressed or not for 72 h. Then, proline was quantified at the indicated time points (**A**). Additionally, as shown in B, two-day-old mycelia were either pre-treated or not with H_2_ for 24 h and then were stressed or not for 24 h. Stressed mycelia were either subjected or not to 500 μM SNP, 500 μM old SNP, or 500 μM cPTIO alone or in the indicated combinations for 30 min after H_2_ treatment. Afterward, proline was measured (**B**). Different letters indicate significant differences between treatments at the indicated time points in A or after 24 h in B according to one-way ANOVA with multiple comparison using Tukey’s test at *p* < 0.05.

Proline participates in the non-enzymatic antioxidation processes in the cells under metal stresses [73]. Thus, HPLC was used to assess proline content in *G. lucidum* mycelia under H_2_ and Cd treatments. As shown in Figure 7A, proline content was progressively increased over 72 h of Cd stress. The samples pre-treated with H_2_ showed significantly higher proline content than Cd stress alone samples after 24 h of Cd treatment. Then, we evaluated the proline contents after H_2_, SNP, and cPTIO treatment to examine the likelihood link between H_2_/NO and proline. As expected, proline in the mycelia was augmented by either H_2_ or SNP, and a maximum increase was observed under H_2_ and SNP successive treatments (Figure 7B). However, cPTIO severely decreased proline accumulation, and old SNP caused no marked effect. In addition, by comparison to the results of the NO peak at 12 h in the H_2_-treated stressed mycelia (Figure 3C), we inferred that the proline rise might be, at least partially, mediated by the H_2_-induced NO under Cd stress.

Further results showed that exogenous proline brought about a significant alleviation in the Cd-caused inhibition of the mycelial growth diameter by 15.23% and the biomass dry weight by 28.94%. However, proline exhibited no significant effect on the mitigation of Cd accumulation (Supplementary Figure S5).

### 3.9. H_2_-Induced Reestablishment of Redox Homeostasis Was Sensitive to cPTIO

**Figure 8:**
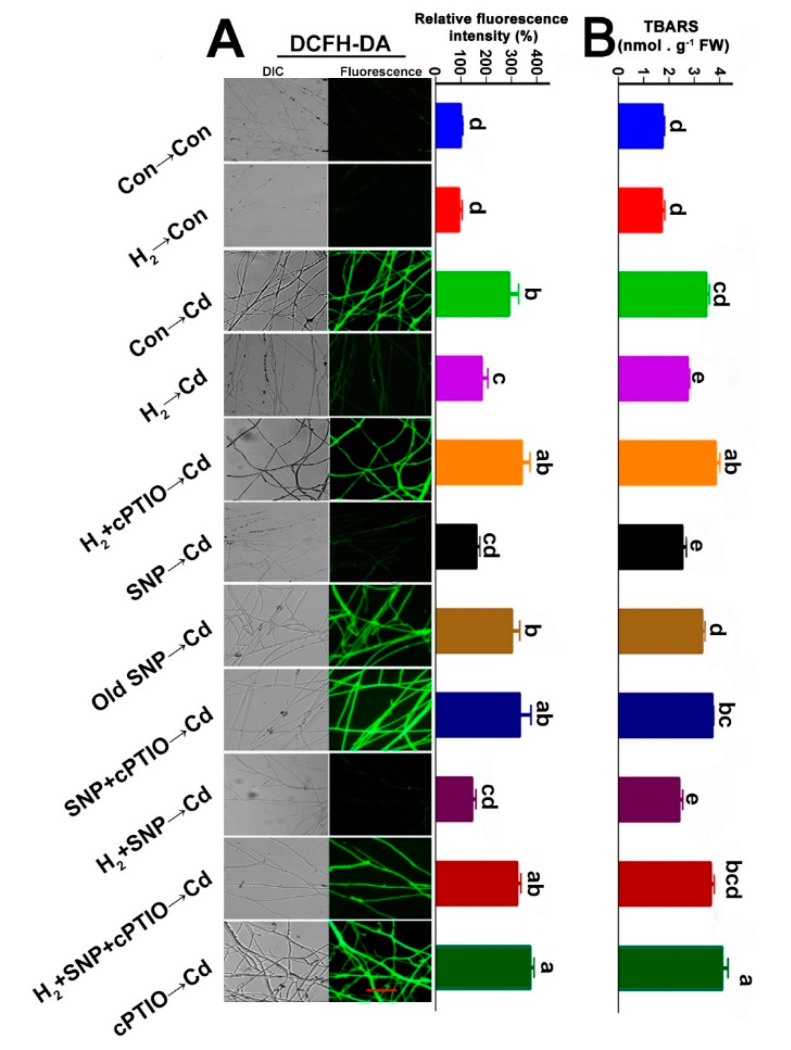
H_2_-reestablished redox balance was sensitive to NO removal. Two-day-old mycelia were either pre-treated or not with 3% H_2_ fumigation for 24 h. Stressed mycelia were either treated or not with 500 µM SNP, 500 µM old SNP, or 500 µM cPTIO alone or in the indicated combinations for 30 min after H_2_ pre-treatment. Then, mycelial discs from the different treatments were cut and grown on either stressed or non-stressed solid CYM for 5 days. Then, the mycelia were loaded with DCFH-DA for ROS analysis (**A**). Scale bar = 100 μm. TBARS contents were also determined (**B**). Mycelia without chemicals were used as control (Con → Con), while H_2_ alone treated mycelia were represented as H_2_ → Con. Bars with different letters are significantly different at *p* < 0.05 according to one-way ANOVA with multiple comparison using Tukey’s test.

Given the knowledge that both cysteine and proline have a powerful influence on oxidative stress, we detected ROS accumulation after H_2_, SNP, cPTIO, and/or Cd treatments (Figure 8). Cd-stressed mycelia showed extensive ROS fluorescence intensities; however, lower ROS were observed in the H_2_- and/or SNP-treated stressed mycelia. When cPTIO was added alone or after H_2_ and/or SNP, it prevented the previous observations. Moreover, TBARS contents exhibited similar trends (Figure 8B). These results revealed that NO contributed to the H_2_-elicited mitigation of the Cd-caused oxidative stress.

The role of the H_2_-induced NO in the re-establishment of redox homeostasis under Cd stress was also confirmed by applying Na_2_WO_4_. Similar to the NO and/or cPTIO elicited responses (Figure 8A,B), the H_2_-elicited decline in ROS and TBARS was reversed by administering Na_2_WO_4_, which could inhibit the NR activity, and consequently reversed the H_2_-induced mitigation (Supplementary Figure S6A,B). Collectively, these results signify that ROS and TBARS were regulated by the H_2_ signaling, which was mediated by NR-related NO functioning under Cd stress.

### 3.10. The NO Scavenger cPTIO Reversed the H_2_-Induced Regulation of the Activities and Related Transcripts of the Antioxidant Enzymes

**Figure 9:**
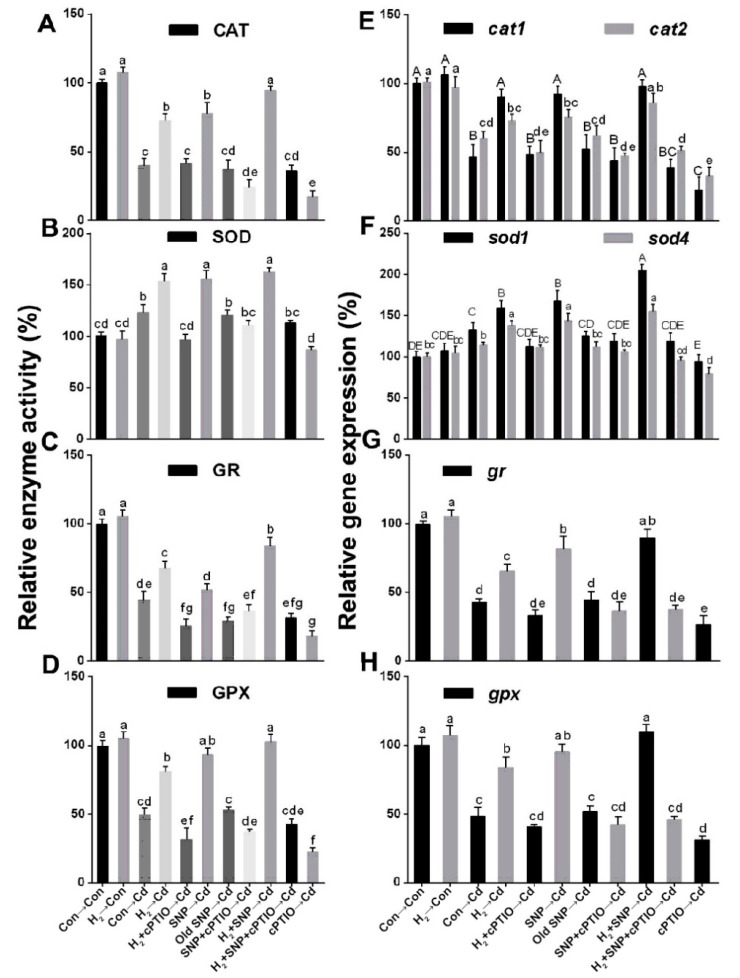
Alterations in the activities of the enzymes CAT, SOD, GR, and GPX and the levels of the corresponding transcripts. Two-day-old mycelia were either pre-treated or not with 3% H_2_ for 24 h. Stressed mycelia were either pre-treated or not with 500 µM SNP, 500 µM old SNP, or 500 µM cPTIO for 30 min alone or in the indicated combinations after H_2_ pre-treatment. Then, mycelial discs from different treatments were grown on stressed or non-stressed solid CYM for 12 or 24 h, respectively. Enzymes’ activities were detected in the 24 h Cd stressed mycelia (**A**–**D**). Corresponding transcripts’ levels were also determined in the 12 h stressed mycelia (**E**–**H**). Mycelia without chemicals were used as the control for each analysis (Con → Con), while H_2_ alone treated mycelia were represented as H_2_ → Con. Bars with different letters are significantly different at *p* < 0.05 according to one-way ANOVA with multiple comparison using Tukey’s test.

The activities of the antioxidant enzymes and their respective gene expression levels were also measured to confirm the NO role in the H_2_-induced effects. After mycelia were exposed to Cd stress for 24 h, the relative activities of CAT, GR, and GPX decreased markedly by 60.07%, 55.40%, and 50.35%, respectively; however, an increase was noticed in SOD by 22.12% relative to the control unstressed mycelia (Figure 9A–D). H_2_ or SNP treatment significantly increased all the antioxidant enzyme activities under Cd stress, and the highest rise was detected under the H_2_ and NO successive treatments by 136.58%, 31.76%, 88.45%, and 107.24% in CAT, SOD, GR, and GPX enzymes, respectively, relative to the Cd alone stressed samples. However, cPTIO reversed the previous responses, and old SNP had no significant effect. Furthermore, the corresponding gene expression levels showed similar tendencies (Figure 9E–H).

## 4. Discussion

Besides the broad application of HRW in medicine and agriculture, it was also utilized to alleviate stresses on fungi, namely mushrooms. However, the water content in HRW may alter the pH and humidity level in the fungi cultivation area, not to mention the possible inconvenience of its application at a large scale on the solid-state cultures of the macroscopic fungi. These reasons make it a less appropriate form. So, we adopted the hydrogen fumigation method in different concentrations on *Ganoderma lucidum* and used 50% HRW as a positive control. Interestingly, we found that 3% H_2_ fumigation initiated a higher alleviation effect of the Cd-elicited inhibition in mycelial growth diameter and biomass dry weight and a lesser Cd accumulation relative to the 50% HRW.

Previous studies on fungi showed the beneficial role of exogenous H_2_ in alleviating different stresses on *Hypsizygus marmoreus* [21], besides the amelioration effects of exogenous NO, individually, on different stresses such as Cd stress on *G. lucidum* [56] and heat shock in *Pleurotus eryngii* [35]. Thus, we investigated a possible plant-like link between the two gaseous signaling molecules, H_2_ and NO, in the fungus *G. lucidum* under Cd stress. Interestingly, our experiments suggested that NO might be involved in the H_2_-motivated alleviation of Cd stress in *G. lucidum* mycelia. We reached this conclusion by analyzing the NO content under 3% H_2_ fumigation pre-treatment. H_2_ strengthened NO release over 72 h of Cd treatment, with a peak at 12 h of Cd exposure (Figure 3C). When the exogenous NO-releasing compound, SNP alone, was added before Cd stress, it also elicited NO release (Figure 4C–E). However, when cPTIO was added, it not only impaired the release of endogenous NO but also reversed both the H_2_-induced amelioration of the mycelial growth inhibition triggered by Cd stress (Figure 4A) and the reduction in heavy metal accumulation (Figure 4B), suggesting the possible participation of NO in the H_2_-induced mitigation response in *G. lucidum* mycelia.

In more detail, we recognized that NR is the source of NO production in the H_2_-pretreated Cd-stressed mycelia. The participation of NR was confirmed by the addition of sodium tungstate (Na_2_WO_4_), an inhibitor of NR alone or in combinations with H_2_. Interestingly, Na_2_WO_4_ reversed the H_2_-elicited amelioration responses in *G. lucidum* (Figure 5). These results are in agreement with a plant study that showed that the osmotic stress tolerance mediated by the exogenous H_2_-elicited NO was prevented in the NR mutant [38]. Cumulatively, the enhancement of NR activity, and hence the amplified NO release in the H_2_-treated stressed mycelia, along with the reversion of H_2_-induced mitigation by the NO scavenger or the NR inhibitor, altogether confirm that the NR-dependent NO evolution was, at least partially, involved in the H_2_-triggered *G. lucidum* tolerance to Cd stress. A recent study showed similar findings where H_2_-enhanced NR-dependent NO alleviated the toxic effects of Cd stress in *Brassica campestris* [39]. More recently, H_2_-induced lateral root formation in tomato seedlings through increasing NR-dependent NO [40]. Based on the above findings, besides many reports of the NO’s regulatory role under stress in fungi [41,74], we concluded that the link between H_2_ and NO is responsible for the H_2_-initiated amelioration of Cd stress in *G. lucidum*. Nevertheless, few conflicting studies revealed an interaction between H_2_ and NO in which the H_2_-induced response was achieved by downregulating NO [60,75]. These contrasting responses may indicate the intricate interactions of these signaling pathways in the different biological systems.

ROS regulation is a key mechanism of the H_2_-initiated alleviation responses to stresses in many animal and plant studies [30]. In fungi, HRW helped the regeneration of *H. marmoreus* mycelia stressed by scratching, improved fruiting body development [76], and prolonged the post-harvest period [22]. The related mechanism was the enhancement of the antioxidant system and redox homeostasis, yet the intermediate signals underlying H_2_ are still largely unknown. Our results illustrated that H_2_ and/or NO reduced ROS and TBARS in the stressed mycelia, and these responses were reversed by the NO scavenger, cPTIO (Figure 8), and the NR inhibitor, Na_2_WO_4_ (Supplementary Figure S6). The above evidence suggests that NO might act downstream of H_2_ in mitigating the ROS abundance elicited by Cd stress in *G. lucidum* (Figure 8). Furthermore, the activities of the antioxidant enzymes CAT, SOD, GPX, and GR and the respective amounts of transcripts *cat1, cat2, sod1, sod4, gr, and gpx* were regulated by H_2_ and/or NO treatments under stress. However, this regulatory effect was blocked by cPTIO (Figure 9). Together with the phenotypic evidence, which showed reversion of the H_2_ ameliorating effect by cPTIO (Figure 4) or Na_2_WO_4_ (Figure 5) addition, we conclude that the H_2_-induced NO might act upstream of the antioxidant system in alleviating Cd stress in *G. lucidum.* The above results are consistent with previous plant and animal findings, indicating a common mechanism in ROS regulation that involves the interaction of H_2_ and NO signals.

Cysteine (Cys) is a thiol-containing amino acid and acts as an indispensable precursor of glutathione (GSH) [77]. In this study, our results showed positive effects of H_2_ on Cys and GSH contents under Cd stress (Figure 6). The time course of Cys levels showed that H_2_ elicited the recovery of the Cd-elicited decline in the Cys levels noticeably after 18 h. This suggested that Cys acted downstream of NO. This signaling pathway was reported in a previous study [72]. Results revealed that H_2_-induced NO mediated an elevation in both Cys and GSH contents under Cd stress. However, this response was reversed by cPTIO, confirming the role of the H_2_-induced NO in the restoration of Cys and GSH levels. The H_2_- and/or NO-induced increase in GSH contents are in harmony with our previous experiments, which showed marked increases in GR and GPX enzymes and transcripts under the same treatments. Some studies suggested the participation of exogenous Cys in the enhancement of heavy metal tolerance in plants [78,79]. Similarly, in our experiments, Cys could alleviate the Cd-caused mycelial growth inhibition, rescue GSH decline, and ameliorate Cd accumulation (Supplementary Figure S4). However, these responses were blocked by the GSH synthesis inhibitor, BSO, suggesting the role of Cys-mediated GSH enhancement in the Cd stress alleviation in *G. lucidum*. This role of GSH in heavy metals chelation and amelioration of the heavy metal toxicity was confirmed individually or through the formation of phytochelatin in previous studies in yeast, protists, and plants [80].

Another significant molecule, proline, participated in the mitigation of different stresses in many plants [81,82,83] and some microorganisms [73], as it protected *Colletotrichum trifolii* fungus against different stresses such as UV light, salt, heat, hydrogen peroxide, and paraquat. Our results revealed that H_2_ fumigation caused a significant increase in proline level after 24 h of Cd stress on *G. lucidum* (Figure 7A), which followed the NO signal peak (Figure 3C). This indicates that NO might act upstream of proline under Cd stress in *G. lucidum.* A previous study suggested that NO-mediated proline aided the *Arabidopsis*’s resistance to cold stress [58]. NO also mediated the accumulation of proline under copper stress in the alga *Chlamydomonas reinhardtii* [84]. Similarly, our experiments revealed that NO alone or in combination with H_2_ pre-treatment enhanced proline accumulation; however, the NO scavenger, cPTIO, severely reduced it relative to the Cd alone stress treatment (Figure 7B). This is consistent with a previous study that suggested that the H_2_-triggered NO signal elevated proline levels by enhancing its synthesis and decreasing its degradation, contributing to osmotic stress tolerance in alfalfa [85].

Proline was also administered under stress to further investigate its participation. Additionally, it significantly alleviated the Cd-triggered inhibition in the mycelial growth diameter and the mycelial biomass dry weight (Supplementary Figure S5A). Cumulatively, previous results suggest that the H_2_-initiated augmentation of proline, intermediated by NO might, at least partially, participate in the amelioration of Cd stress in *G. lucidum*. Nevertheless, proline did not lessen Cd accumulation (Supplementary Figure S5B), implying that it works through other pathways rather than Cd sequestration in *G. lucidum*, possibly through antioxidation mechanism, and this requires further investigation.

**Figure 10:**
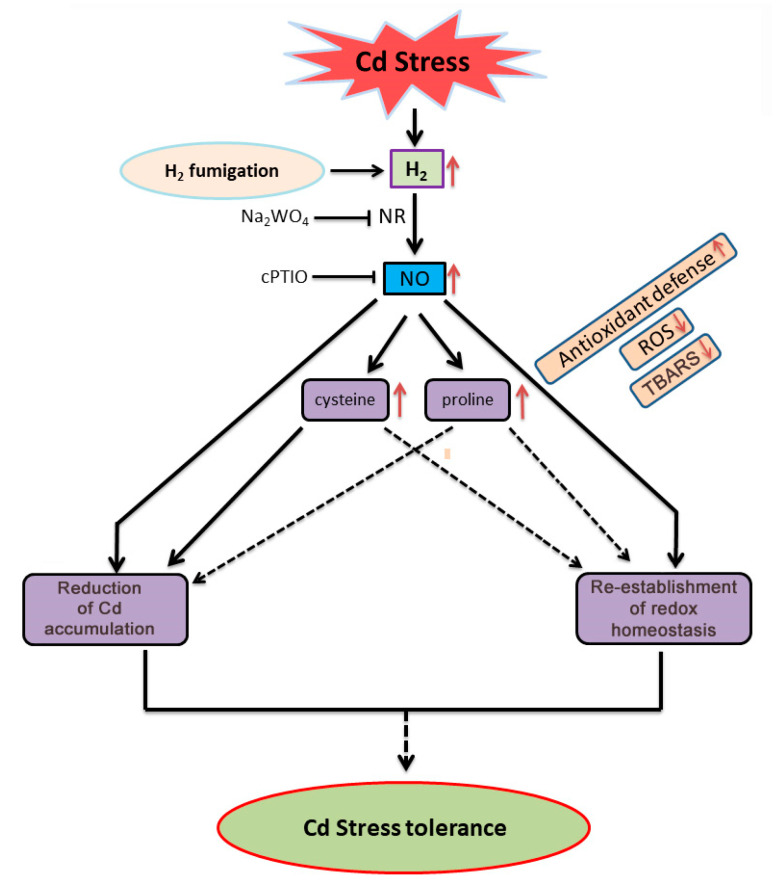
A model illustrating the participation of NO and the possible involvement of cysteine and proline in the H_2_-induced alleviation of Cd stress on *G. lucidum.* In this model, the solid arrows and T bars indicate data supported by our own experiments. The dashed arrows and T bars indicate hypothetical steps or findings from previous studies. T bar represents the inhibitory or scavenging effect.

Overall, our report provides the underlying mechanism of the H_2_-induced mitigation of Cd stress in *G. lucidum,* mainly through the NO-mediated regulation of ROS homeostasis, the reduction in Cd accumulation, and possibly, the increase in cysteine and proline accumulation (Figure 10). Moreover, this is the first report on fungi that reveals the advantages of H_2_ fumigation in stress alleviation.

## 5. Conclusions

The link we found between H_2_ and NO in the alleviation of Cd stress on *G. lucidum* will provide new insights into the mechanisms of H_2_ stress alleviation in *G. lucidum*, and this may also be further deployed on the molecular level in other valuable pathways beyond enhancing stress mitigation in *G. lucidum* and other fungi. Most importantly, our report provides a convenient model for hydrogen application on fungi (hydrogen fumigation), which brought about a higher response than HRW in alleviating Cd heavy metal stress. This model could be harnessed on a large scale in the economically important macroscopic fungi farms by fumigating fungi at a suitable rate through perforated pipelines connected to a large hydrogen source, aiming to achieve higher stress alleviation and higher productivity, similar to our in vitro findings.

## Data Availability

All data generated or analyzed during this study are included in this published article and its Supplementary Materials.

## Supplementary Materials

The following are available online at https://www.mdpi.com/article/10.3390/jof8010010/s1, Table S1: The sequences of primers used for RT-qPCR in *Ganoderma lucidum*. Figure S1: Effect of different Cd concentrations on mycelial growth of *G. lucidum*; Figure S2: Screening for the optimum HRW concentration for the alleviation of 1.75 mM CdCl_2_ stress on *G. lucidum.* Figure S3: H_2_-fumigation pre-treatment activated the antioxidant enzyme activities and their corresponding transcript levels under oxidative stress caused by Cd stress. Figure S4: Cd-elicited negative effects on mycelial growth, GSH and Cd accumulation was rescued by cysteine. Figure S5: Proline alleviated the growth inhibition caused by Cd stress but did not mitigate Cd accumulation. Figure S6: H_2_-reestablished redox balance was sensitive to sodium tungstate, an inhibitor of NR enzyme activity. Figure S7: H_2_, HRW, SNP, NO’s scavenger, cPTIO, Na_2_WO_4_ or proline had no significant effect on mycelial growth or NO content under normal conditions.
