# Supplementary material for: The Importance of Nitric Oxide as the Molecular Basis of the Hydrogen Gas Fumigation-Induced Alleviation of Cd Stress on Ganoderma lucidum
Source: J Fungi (Basel). 2021 Dec 23;8(1):10. doi: 10.3390/jof8010010 (PMC8780922; doi:10.3390/jof8010010)
Supplement: Supplementary file 1 [file jof-08-00010-s001.zip › jof-1496758-supplementary.pdf]

SUPPLEMENTARY TABLES AND FIGURES

Supplementary Table S1. The sequences of primers and reference gene used for RT-qPCR in *Ganoderma lucidum*

Primer	Sequence (5′ to 3′)
18S-F	TATCGAGTTCTGACTGGGTTGT
18S-R	ATCCGTTGCTGAAAGTTGTAT
cat1-F	TACGGTATTCAGCTCTTGT
cat1-R	TGTTGTAACGGAACTTCTC
cat2-F	GAAGAGGCGATCACTACCG
cat2-R	GAAACCGATGCCAGGAACA
sod1-F	ATCGCCGTCTTCGTCGTTT
sod1-R	GTGACTGGTGCGGTAGGGA
sod4-F	CTCACCGCGACGCGATTAC
sod4-R	CCCTTGCCCTCAGACTTGG
gr -F	CGGGACTTGCGTGAATGTTG
gr-R	TTCTTTGGCACGCATCCTGT
gpx-F	ATGTCCGACGCAGGATTCTACT
gpx-R	GGAAGCCGAGAATGACGAAG

Supplementary Figure S1

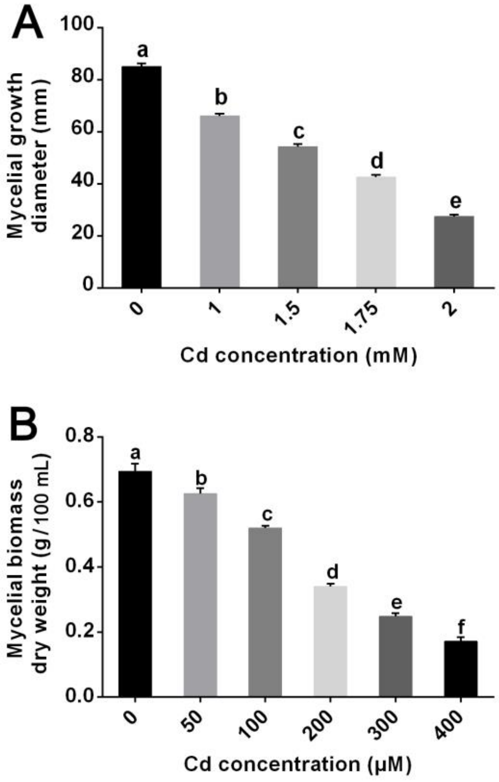

Supplementary Figure S1. Effect of different Cd concentrations on mycelial growth of *Ganoderma lucidum*. Mycelia were grown on solid PDA media for 7 d (solid seed). Then 6-mm-diameter mycelial discs from the solid seed were transferred to solid CYM media amended with $CdCl_2$ at different concentrations 0, 1, 1.5, 1.75, or 2 mM and incubated for 8 d at 28°C in the dark. Meanwhile, sets of 8 mycelial discs were cut from the solid seed and each set was inoculated into 100 mL liquid CYM media with $CdCl_2$ at different concentrations 0, 50, 100, 200, 300, or 400 μM and grown for 8 d in shaking incubator at 28°C in the dark. After that, the diameter of mycelial growth (A) or the mycelial biomass dry weight (B) was determined. Values are the means ± SD of three independent experiments with at least three biological replicates for each. Bars with different letters are significantly different at $P < 0.05$ according to one-way ANOVA with multiple comparison using Tukey's test.

Supplementary Figure S2

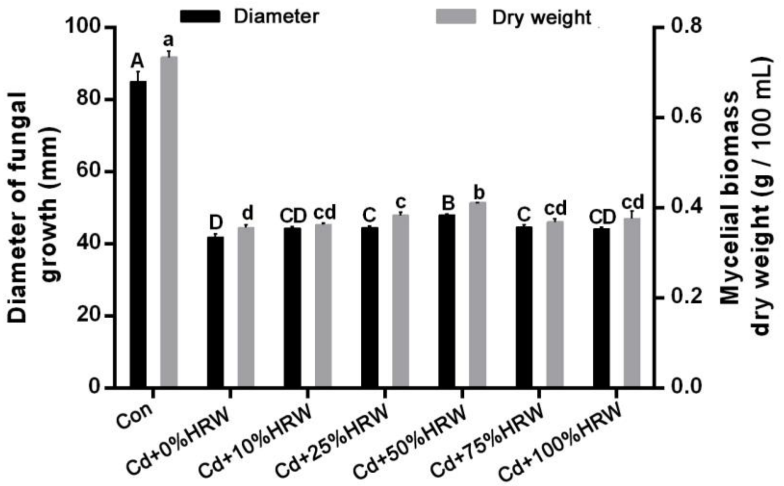

Supplementary Figure S2. Screening for the optimum HRW concentration for the alleviation of Cd stress on *G. lucidum*. Mycelial inocula were grown on solid CYM media for 2 d. On the 3rd day, mycelia were pre-treated with 10%, 25%, 50%, 75% or 100% HRW for 24 h or not treated with HRW altogether. On the 4th day, 6-mm-diameter mycelial discs from HRW treated or non-treated mycelia were grown on solid CYM media amended with 1.75 mM $CdCl_2$ for 8 d. Meanwhile, other mycelia were grown on solid CYM without $CdCl_2$ for 8 d (Con). Afterward, the diameters of the mycelial growth were detected (left). Simultaneously, on the 4th day, sets of 8 identical mycelial discs were cut from the HRW-treated or non-treated mycelia, and each set was inoculated into Cd-stressed liquid CYM, and then were cultivated in shaking incubator for 8 d in the dark. Then the biomass dry weight values were immediately measured (right). Mycelia without chemical treatments were used as control (Con). Values are the means ± SD of three independent experiments with at least three biological replicates for each. Bars with different letters are significantly different at $P < 0.05$ according to one-way ANOVA with multiple comparison using Tukey's test.

Supplementary Figure S3

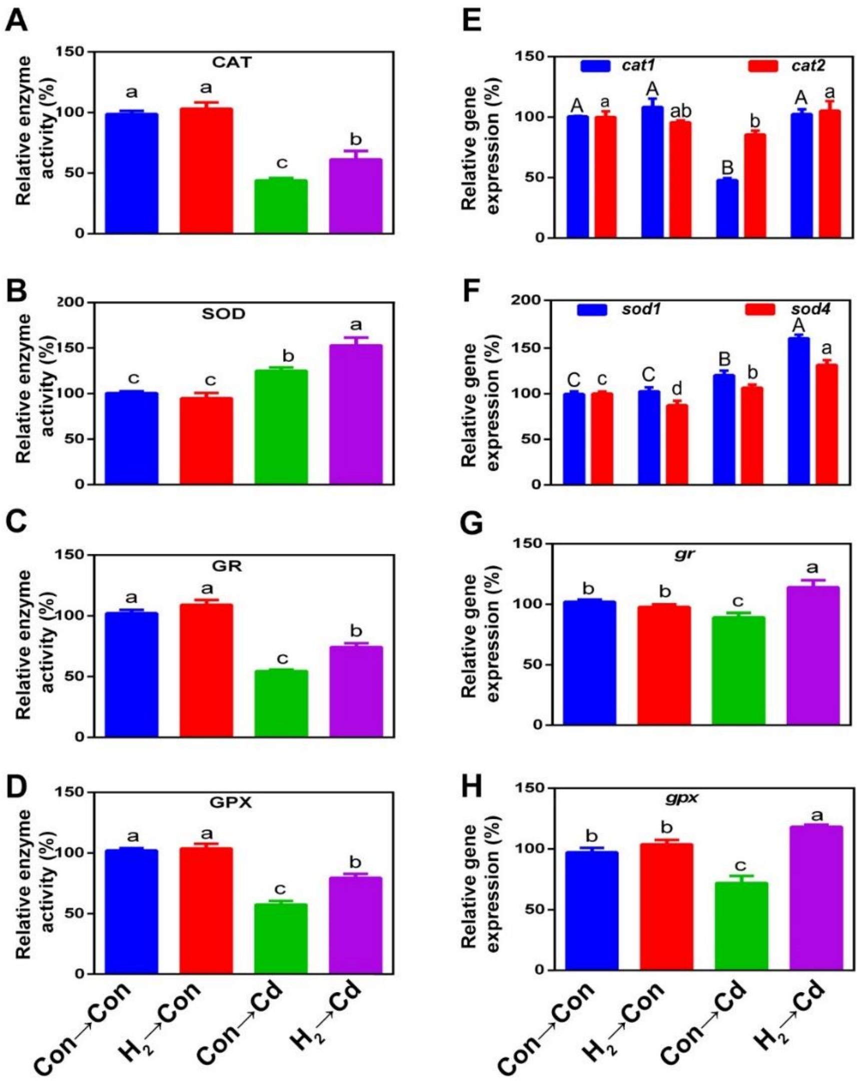

Supplementary Figure S3. H_2-fumigation enhanced the antioxidant enzyme activities and their corresponding transcript levels under oxidative stress caused by Cd stress. Two-day-old mycelia were pre-treated or not with 3% H_2 for 24 h; Then, 6-mm-diameter mycelial discs from these mycelia were grown on solid CYM media (normal condition) or $CdCl_2$–containing solid CYM (stress treatment) for 12 or 24 h, then the enzymatic activities of CAT, SOD, GR, and GPX were measured in the 24 h-stressed or unstressed mycelia (A–D). The transcript levels of these antioxidant enzymes were also measured in the 12 h stressed or unstressed mycelia (E-H). The transcript levels detected by RT-qPCR were represented as values relative to the control sample. Mycelia without chemicals were used as control. Values are means ± SD of three independent experiments with three biological replicates for each. Bars with different letters are significantly different at $P < 0.05$ according to one-way ANOVA with multiple comparison using Tukey's test.

Supplementary Figure S4

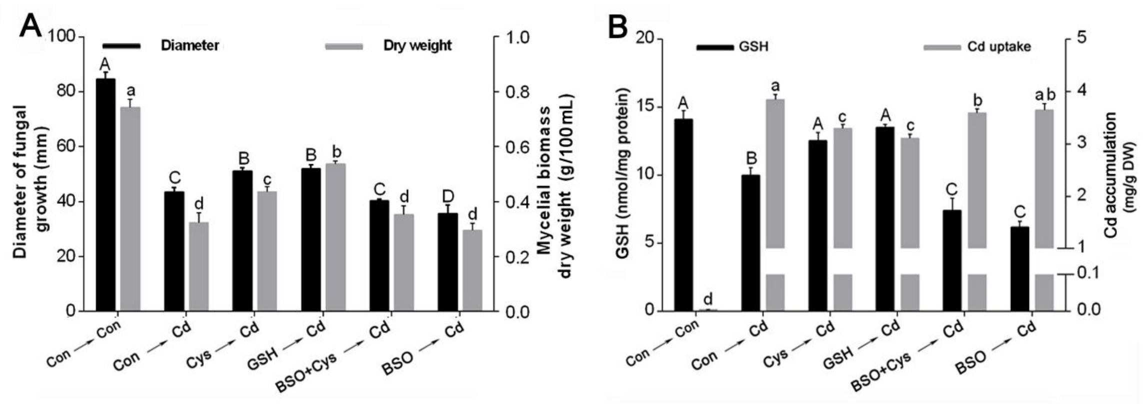

Supplementary Figure S4. Exogenous cysteine (Cys) alleviated the growth inhibition caused by Cd stress on *G. lucidum*. Mycelia were cultured on Solid CYM media for 2 d. On the 3rd day, mycelia were either pre-treated or not with 300 μM Cys or 325 μM GSH, each for 24 h. Meanwhile, other samples were subjected to 2.5 mM BSO for 1 h alone or before Cys treatment. On the 4th day, 6mm diameter mycelial discs from these different treatments were grown on stressed or non-stressed solid or liquid CYM media for 8 d. Afterward, the mycelial growth diameter (A; left) and biomass dry weight (A; right) values were determined. Simultaneously, on the 4th day, other sets of mycelia from the different treatments were either stressed or not for 24 h or 5 d, then were analyzed for GSH and Cd contents, respectively (B). Mycelia without chemicals were represented as Con → Con. Values are means ± SD of three independent experiments with three biological replicates for each. Bars with different letters are significantly different at $P < 0.05$ according to one-way ANOVA with multiple comparison using Tukey's test.

Supplementary Figure S5

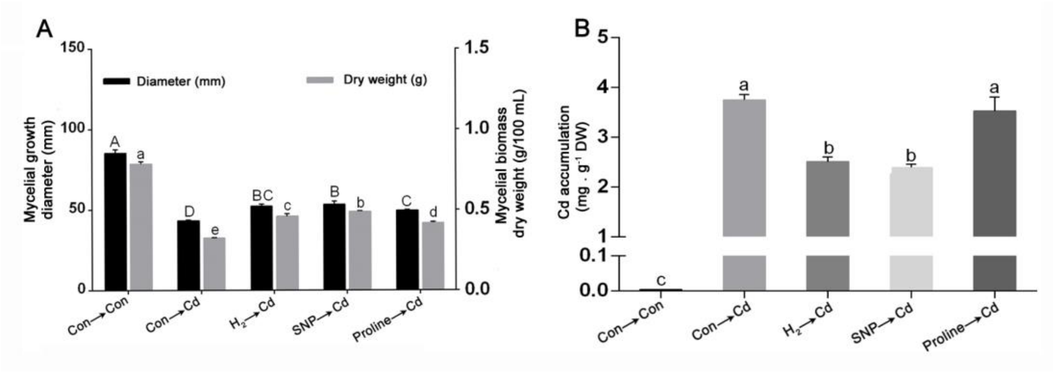

Supplementary Figure S5. Proline alleviated the growth inhibition caused by Cd stress on *G. lucidum*. Mycelia were cultured on solid CYM media for 2 d. Then on the 3rd day, mycelia were either pre-treated or not with 3% H_2 fumigation for 24 h. On the 4th day, 6-mm-diameter mycelial disc(s) from the H_2 treated or non-treated mycelia were grown on stressed or non-stressed solid or liquid CYM media for 8 d then the mycelial growth diameter (A; left) or mycelial biomass dry weight (A; right) values were determined. Simultaneously, on the 4th day, another set of mycelial discs were grown for 5 d on stressed or non-stressed solid CYM, then Cd uptake was analyzed (B). In both (A) and (B), the non-fumigated stressed samples were either pre-treated or not with 500 μM SNP for 30 min, or 10 μM Proline for 12 h before Cd exposure. Mycelia without chemicals were represented as Con → Con. Values are means ± SD of three independent experiments with three biological replicates for each. Bars with different letters are significantly different at $P < 0.05$ according to one-way ANOVA with multiple comparison using Tukey's test.

Supplementary Figure S6

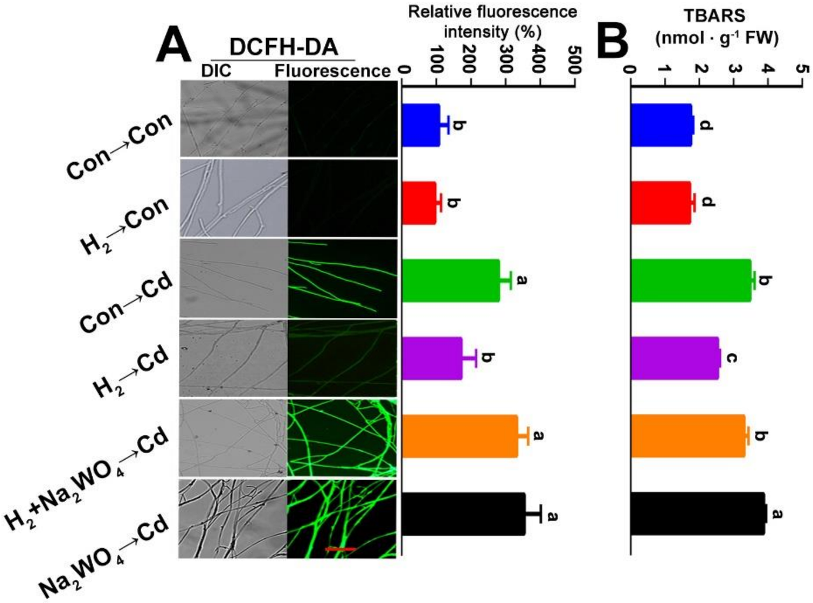

Supplementary Figure S6. H_2-induced reestablishment of redox homeostasis was susceptible to Na_2WO_4. 2 d old mycelia were either pre-treated or not with 3% H_2 fumigation for 24 h. Stressed mycelia were either pre-treated or not with 500 μM Na_2WO_4 for 30 min alone or after H_2 pre-treatment. Then, 6-mm-diameter mycelial discs from the different treatments were cut and grown on solid CYM media (normal condition) or $CdCl_2$–containing solid CYM (stress treatment) for 5 d. Afterward, the mycelia were loaded with DCFH-DA for ROS fluorescence analysis (A). Scale bar = 100 μm. DIC, differential interference contrast. Meanwhile, TBARS contents were also determined (B). Mycelia without chemicals were used as the control for each analysis Con → Con, while H_2 alone treated mycelia were represented as H_2 → Con. Values are the means ± SD of three independent experiments with at least three biological replicates for each. Bars with different letters are significantly different at $P < 0.05$ according one-way ANOVA with multiple comparison using Tukey's test.

Supplementary Figure S7

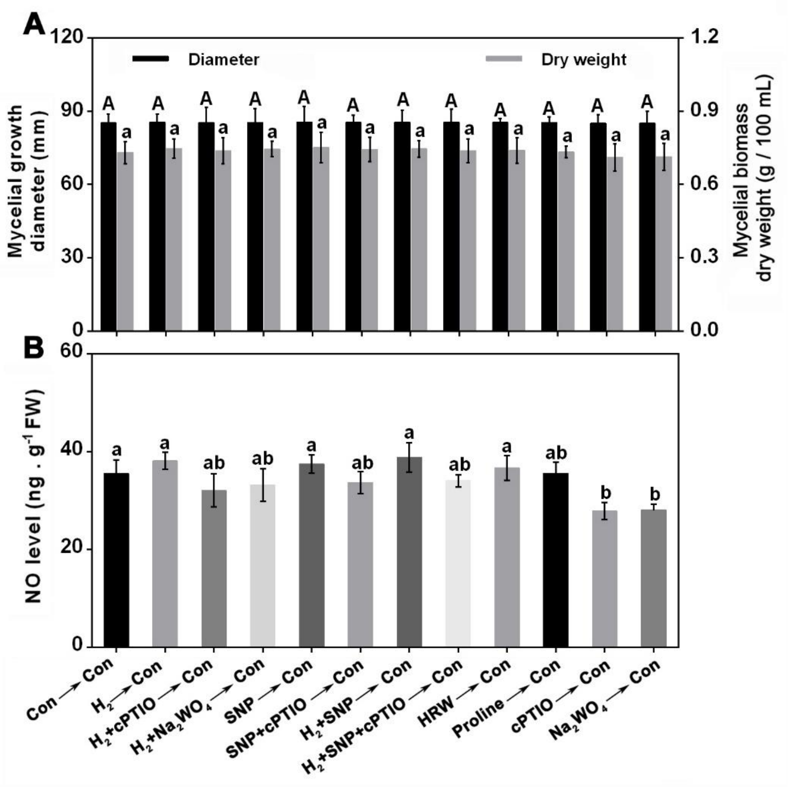

Supplementary Figure S7. H_2, HRW, SNP, NO's scavenger, cPTIO, Na_2WO_4 or proline had no significant effect on mycelial growth or NO content under normal conditions. Mycelial inocula were grown on solid CYM media for 2 d. On the 3rd day, mycelia were either pre-treated or not with 3% H_2, 50% HRW, each for 24 h; or 10 μM proline for 12 h. H_2 treated or non-treated mycelia were either treated or not with 500 μM SNP, 500 μM cPTIO or 500 μM Na_2WO_4 alone or in the indicated combinations. Mycelial discs from the different treatments were grown for 12 hours or 8 days on solid or liquid CYM media. Values of growth diameter and the biomass dry weight in the eight-day-old mycelia were measured (A). Twelve-hour-old mycelia were analyzed for NO contents by Griess reagent assay (B). Bars with different letters are significantly different at $P < 0.05$ according to one-way ANOVA with multiple comparison using Tukey's test.
